# Supplementary material for: Decapping activators Edc3 and Scd6 act redundantly with Dhh1 in post-transcriptional repression of starvation-induced pathways
Source: bioRxiv. 2024 Aug 28:2024.08.28.610059. Preprint. [Version 1] doi: 10.1101/2024.08.28.610059 (PMC11383670; doi:10.1101/2024.08.28.610059)
Supplement: Supplementary file 1 [file NIHPP2024.08.28.610059v1-supplement-1.pdf]

bioRxiv preprint doi: https://doi.org/10.1101/2024.08.28.610059; this version posted August 28, 2024. The copyright holder for this preprint (which was not certified by peer review) is the author/funder. This article is a US Government work. It is not subject to copyright under 17 USC 105 and is also made available for use under a CC0 license.

SUPPLEMENTARY MATERIAL FOR:

Decapping activators Edc3 and Scd6 act redundantly with Dhh1 in nutrient-replete cells to post-transcriptionally repress starvation-induced pathways

Rakesh Kumar[1#], Fan Zhang[1#], Shreyas Niphadkar[3], Chisom Onu[2], Anil Kumar Vijjamarri[1], Miriam L. Greenberg[2], Sunil Laxman[3], and Alan G. Hinnebusch[1]

bioRxiv preprint doi: https://doi.org/10.1101/2024.08.28.610059; this version posted August 28, 2024. The copyright holder for this preprint (which was not certified by peer review) is the author/funder. This article is a US Government work. It is not subject to copyright under 17 USC 105 and is also made available for use under a CC0 license.

SUPPLEMENTARY TABLES

Table S1. Yeast strains employed.

Strain	Genotype	Source
255	*MATa his3Δ1 leu2Δ0 met15Δ0 ura3Δ0 edc3Δ::kanMX4*	Research Genetics
HFY114 (W303)	*MATa ade2-1 ura3-1 his3-11,15 trp1-1 leu2-3, 112 can1-100*	(1)
SYY2352	*MATa ade2-1 ura3-1 his3-11,15 trp1-1 leu2-3, 112 can1-100 scd6Δ::kanMX6*	(2)
FZY855	*MATa ade2-1 ura3-1 his3-11,15 trp1-1 leu2-3, 112 can1-100 scd6Δ::hphMX4*	This study
FZY858	*MATa ade2-1 ura3-1 his3-11,15 trp1-1 leu2-3, 112 can1-100 scd6Δ::hphMX4 edc3Δ::kanMX4*	This study
FZY862	*MATa ade2-1 ura3-1 his3-11,15 trp1-1 leu2-3, 112 can1-100 edc3Δ::kanMX4*	This study
H5217/QZY126	*MATa ade2-1 ura3-1 his3-11,15 trp1-1 leu2-3,112 can1-100 dhh1Δ::kanMX*)	(3)
F2181/ BSY3037	*MATa ade2-1 ura3-1 his3-11,15 trp1-1 leu2-3,112 can1-100 pat1Δ::HIS3*)	(4)
F2182/ YFW168	*MATa ade2-1 ura3-1 his3-11,15 trp1-1 leu2-3,112 can1-100 pat1Δ::HIS3 dhh1Δ::kanMX*	(4)
CFY1016	*MATa ade2-1 ura3-1 his3-11,15 trp1-1 leu2-3,112 can1-100 dcp2::HIS3*	(1)

Table S2. Plasmids employed.

Plasmid	Description	Source
YCplac33	s.c. *URA3* vector	(5)
YCplac111	s.c. *LEU2* vector	(5)
pLfz614-7	*EDC3* in YCplac33	This study
pLfz615-5	*SCD6* in YCplac33	This study
pLfz635-5	*EDC3* in YCplac111	This study (in case used)
pLfz636-1	*SCD6* in YCplac111	This study (in case used)
pLGADH2	*ADH2* 5’ non-coding region fused to *lacZ*	(6)
pLG265	*CYC1-lacZ* reporter lacking UAS1 and containing the optimized version of UAS2, UAS2UP1	(7)
pRK4	UAS_{GATA}-*CYC1-lacZ* reporter containing the UAS from *MEP2* modified to contain additional GATA sequences	(8)

bioRxiv preprint doi: https://doi.org/10.1101/2024.08.28.610059; this version posted August 28, 2024. The copyright holder for this preprint (which was not certified by peer review) is the author/funder. This article is a US Government work. It is not subject to copyright under 17 USC 105 and is also made available for use under a CC0 license.

bioRxiv preprint doi: https://doi.org/10.1101/2024.08.28.610059; this version posted August 28, 2024. The copyright holder for this preprint (which was not certified by peer review) is the author/funder. This article is a US Government work. It is not subject to copyright under 17 USC 105 and is also made available for use under a CC0 license.

SUPPLEMENTARY FIGURES

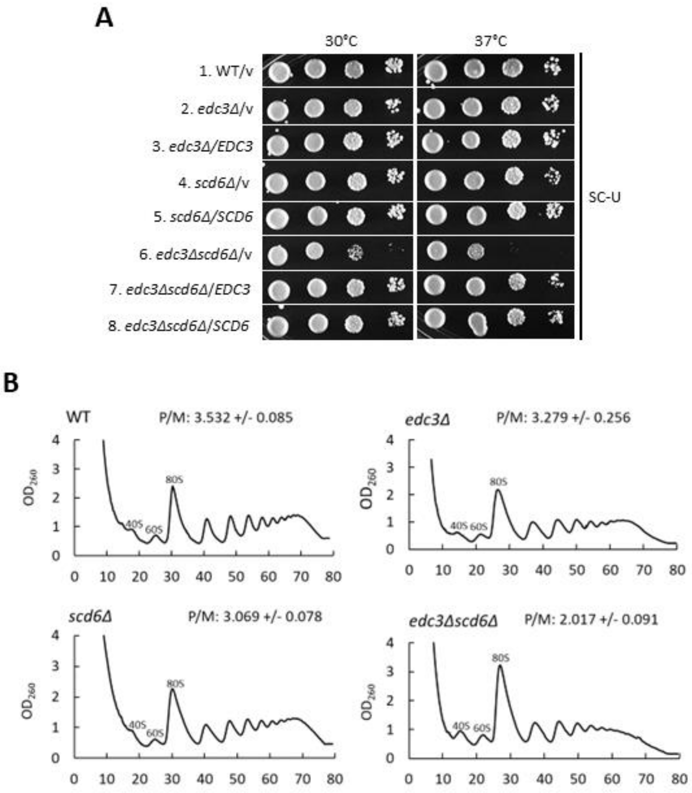

Figure S1. Combining *scd6Δ* and *edc3Δ* mutations confer synthetic reductions in cell growth and polysome assembly. (A) Serial dilutions of WT strain HFY114, *edc3Δ* strain FZY862, *scd6Δ* strain SYY2352, and *scd6Δedc3Δ* strain FZY858 transformed with empty *URA3 CEN* vector YCplac33 or derivatives of this vector containing *EDC3* (pLfz614-7) or *SCD6* (pLfz615-5) were spotted on synthetic complete plates minus uracil (SC-Ura) and incubated at the indicated temperatures.

(B) Polysome profiles of the strains in (A) but lacking plasmids cultured in YPD medium at 30°C to log-phase growth and treated with cycloheximide prior to harvesting to block run-off of elongating ribosomes during cell lysis. Cell extracts were resolved by sedimentation through 10-50% sucrose gradients, and gradients were scanned at 260 nm to yield the indicated tracings.

bioRxiv preprint doi: https://doi.org/10.1101/2024.08.28.610059; this version posted August 28, 2024. The copyright holder for this preprint (which was not certified by peer review) is the author/funder. This article is a US Government work. It is not subject to copyright under 17 USC 105 and is also made available for use under a CC0 license.

Average polysome/monosome ratios (P/M) from 2 biological replicates are shown, with means +/- SEMs.

bioRxiv preprint doi: https://doi.org/10.1101/2024.08.28.610059; this version posted August 28, 2024. The copyright holder for this preprint (which was not certified by peer review) is the author/funder. This article is a US Government work. It is not subject to copyright under 17 USC 105 and is also made available for use under a CC0 license.

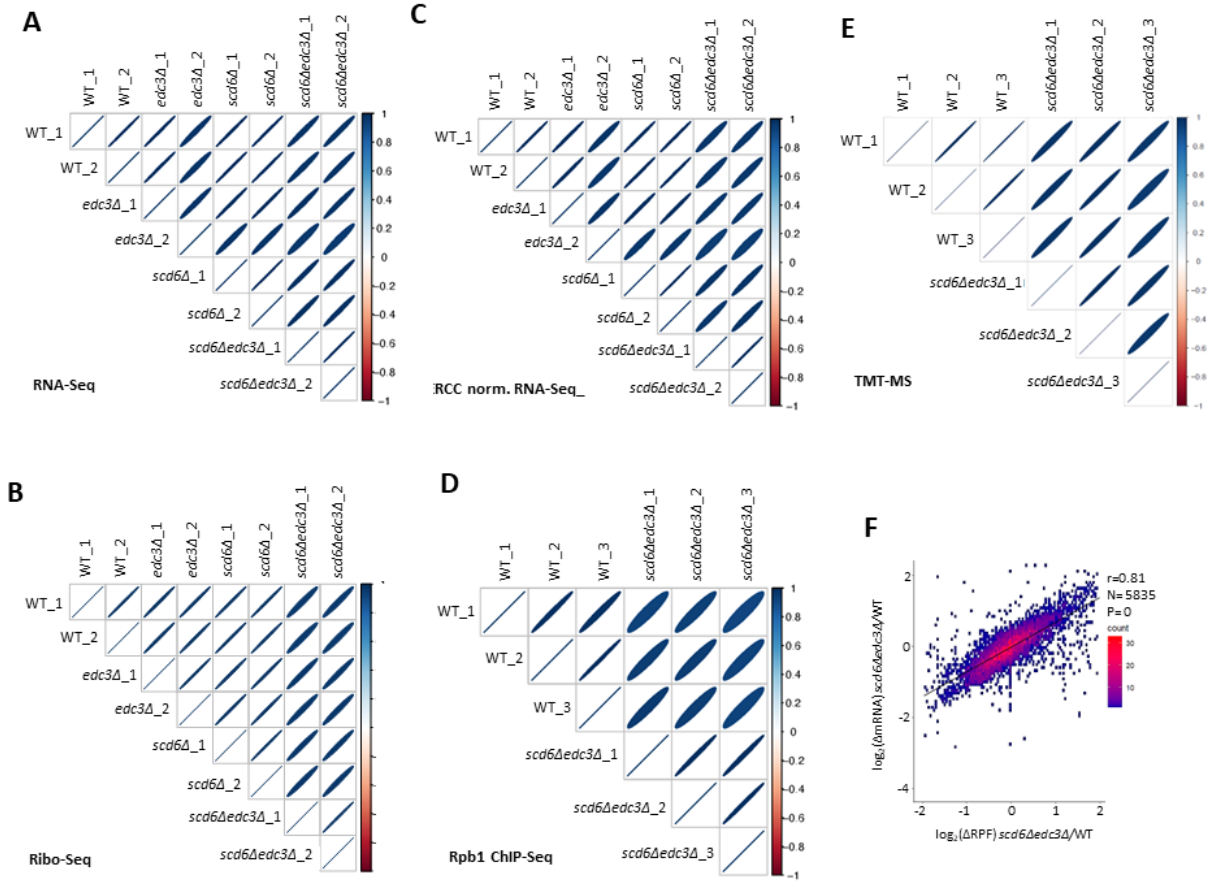

Figure S2. Reproducibility among biological replicates of RNA-Seq, Ribo-Seq, ERCC-normalized RNA-Seq, Rpb1 ChIP-seq, and TMT-MS data. (A) Correlation matrix showing Spearman correlation coefficients calculated for pair-wise comparisons of numbers of RPKM-normalized RNA-Seq reads for all expressed genes among all 8 RNA-Seq libraries generated for 2 biological replicates (_1,_2) of the indicated genotypes. The correlation coefficients between replicates are ≥0.98 and for all comparisons >0.95 with P-values of ≈0. For this and all similar plots below, the eccentricity of the ellipses are scaled parametrically to the correlation value between the two samples. **(B)** Same as (A) but for Ribo-Seq data, with correlation coefficients between replicates >0.995 and for all comparisons >0.96 with P-values of ≈0. **(C)** Same as (A) but for ERCC-normalized RNA-Seq data, with correlation coefficients between replicates >0.97 and for all comparisons >0.94 with P-values of ≈0. **(D)** Same as (A) but for Rpb1 ChIP-Seq data

bioRxiv preprint doi: https://doi.org/10.1101/2024.08.28.610059; this version posted August 28, 2024. The copyright holder for this preprint (which was not certified by peer review) is the author/funder. This article is a US Government work. It is not subject to copyright under 17 USC 105 and is also made available for use under a CC0 license.

comparing 3 normalized occupancies averaged across the CDS of all 5770 expressed genes, with correlation coefficients between replicates >0.98 and for all comparisons >0.91 with P-values of ≈0. **(E)** Same as (A) but for TMT-MS data, comparing $\log_2$ cyclic Loess-normalized exclusive MS1 intensities for all expressed proteins with correlation coefficients between replicates >0.96 and for all comparisons >0.95 with P-values of ≈0. **(F)** Correlation between changes in abundance of RNA vs. RPFs for 5835 expressed transcripts in the *scd6Δedc3Δ* double mutant compared to WT, calculated from RPKM-normalized RNA-Seq and Ribo-Seq reads obtained after combining data from biological replicates, with the Pearson correlation coefficient (r) and P-value of the correlation indicated.

bioRxiv preprint doi: https://doi.org/10.1101/2024.08.28.610059; this version posted August 28, 2024. The copyright holder for this preprint (which was not certified by peer review) is the author/funder. This article is a US Government work. It is not subject to copyright under 17 USC 105 and is also made available for use under a CC0 license.

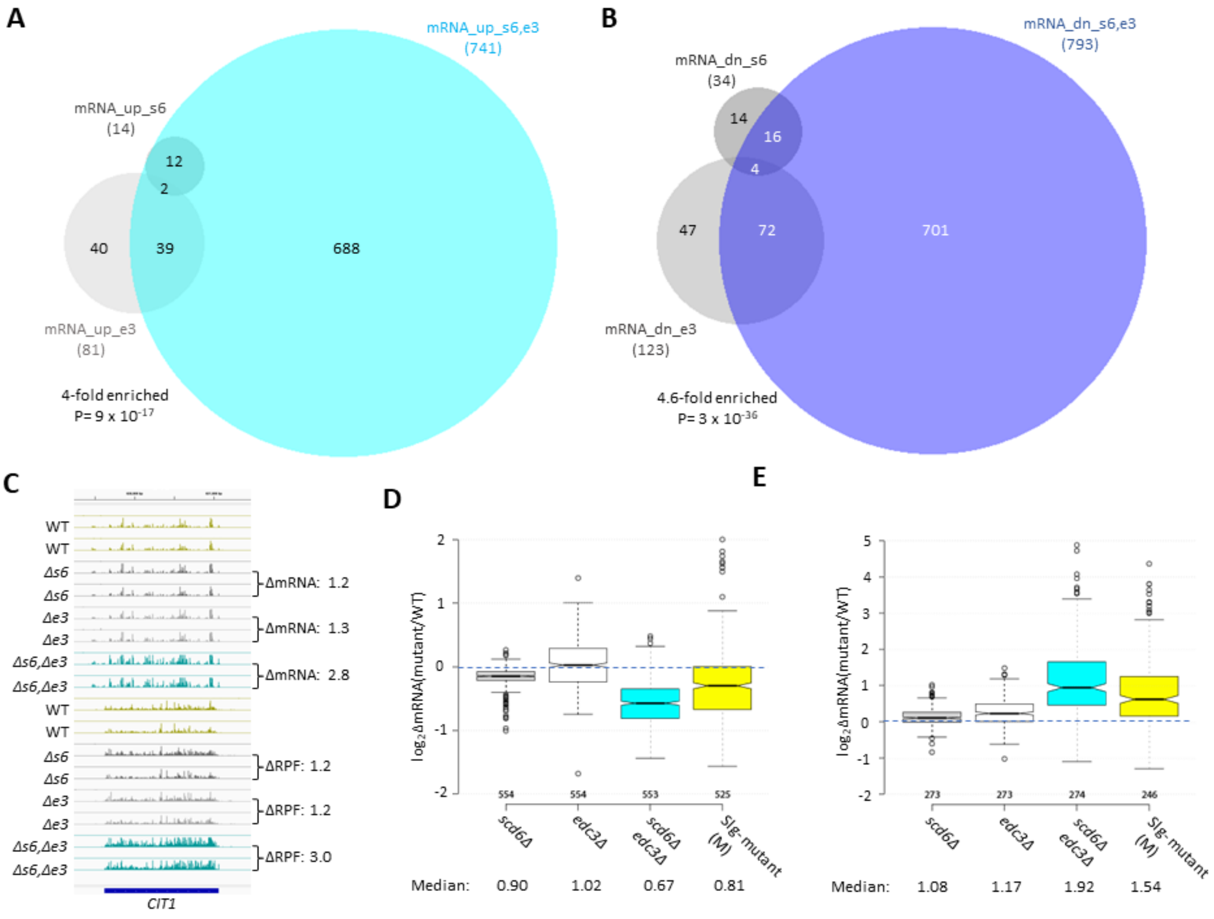

Figure S3. Functional redundancy between Scd6 and Edc3 in controlling mRNA abundance and mobilizing the ESR. (A-B) Venn diagrams of overlaps between all mRNA_up (A) and all mRNA_dn (B) groups identified in *scd6Δ, edc3Δ*, or *scd6Δedc3Δ* mutants vs. WT, with fold-enrichments and P values from the hypergeometric distribution indicated for overlapping sets. **(C)** Gene browser image showing the mRNA (top 16 tracks) and RPF (bottom 16 tracks) reads measured by ribosome profiling for two biological replicates of WT and the indicated mutants for *CIT1*, with fold-changes in mRNA or RPFs between mutant and WT indicated to the right of each track. **(D-E)** Notched box-plot analyses of $\log_2$ changes in mRNA abundance in mutant vs. WT for 585 rESR (C) and 283 iESR (D) mRNAs conferred by *scd6Δ, edc3Δ*, or *scd6Δedc3Δ* mutations and those observed for the slowest-growing yeast deletion mutants (M) analyzed previously (9). Each box

bioRxiv preprint doi: https://doi.org/10.1101/2024.08.28.610059; this version posted August 28, 2024. The copyright holder for this preprint (which was not certified by peer review) is the author/funder. This article is a US Government work. It is not subject to copyright under 17 USC 105 and is also made available for use under a CC0 license.

depicts the interquartile range containing 50% of the data, intersected by the median; the notch indicates a 95% confidence interval (CI) around the median. Median changes (un-logged) are shown at the bottom.

bioRxiv preprint doi: https://doi.org/10.1101/2024.08.28.610059; this version posted August 28, 2024. The copyright holder for this preprint (which was not certified by peer review) is the author/funder. This article is a US Government work. It is not subject to copyright under 17 USC 105 and is also made available for use under a CC0 license.

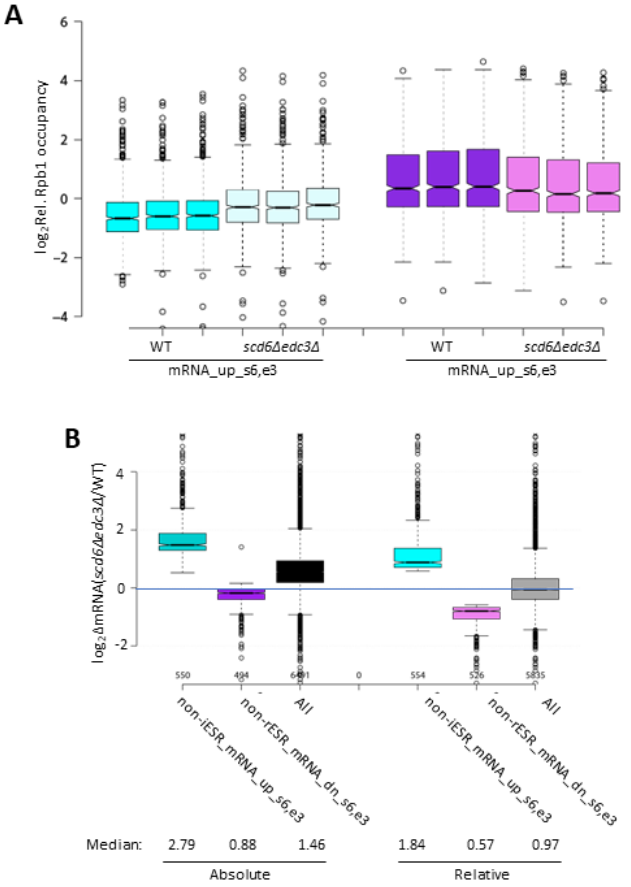

Figure S4. Reproducibility among biological replicates of Rpb1 ChIP-seq data. Log_2 values of relative Rpb1 occupancies averaged over the CDSs for three replicates of WT or *scd6Δedc3Δ* cells for the mRNAs up- or down-regulated by *scd6Δedc3Δ* vs. WT, including ESR transcripts. **(B)** Notched box-plots showing log_2 changes in absolute mRNA abundance from ERCC spike-in normalized RNA-Seq (left) or relative mRNA abundance determined by DESeq2 analysis of RNA-Seq results (right) in *scd6Δedc3Δ* vs. WT cells for all mRNAs or the 554 or 526 non-ESR mRNAs up- or down-regulated, respectively, by *scd6Δedc3Δ* vs. WT.

bioRxiv preprint doi: https://doi.org/10.1101/2024.08.28.610059; this version posted August 28, 2024. The copyright holder for this preprint (which was not certified by peer review) is the author/funder. This article is a US Government work. It is not subject to copyright under 17 USC 105 and is also made available for use under a CC0 license.

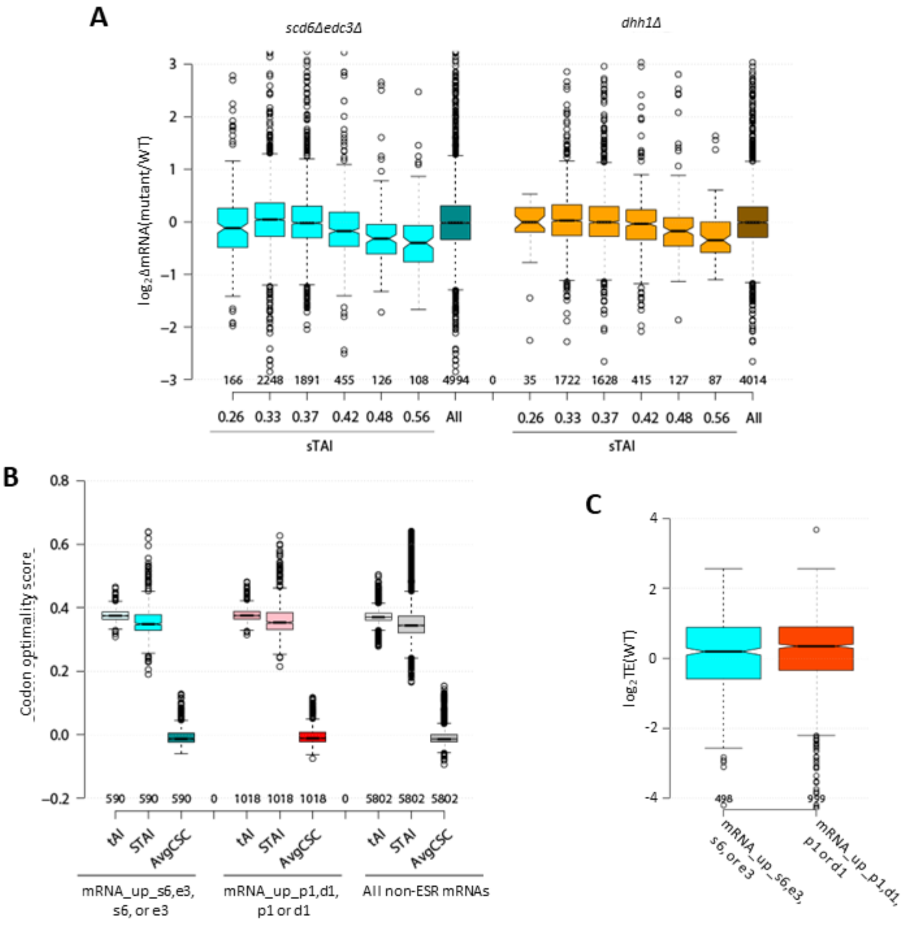

Figure S5. Average median codon optimality scores and average median TE values in WT cells for mRNAs repressed in abundance by Scd6/Edc3 or Dhh1/Pat1. (A) Notched box-plots of $\log_2\Delta$mRNA between *scd6Δedc3Δ* (left, cyan) or *dhh1Δ* (right, orange) vs. WT for 6 bins of all non-ESR mRNAs sorted according to stAI values, designated by the median stAI value for each bin, or for all non-ESR mRNAs (All). **(B)** tAI, stAI, and average CSC values for the 591 non-iESR mRNAs derepressed in abundance by the *scd6Δ, edc3Δ*, or *scd6Δedc3Δ* mutations, the 1018 non-ESR mRNAs up-regulated by the *dhh1Δ, pat1Δ*, or *pat1Δdhh1Δ* mutations, or all 5802 non-ESR mRNAs. **(C)** Log_2 values of TE determined in WT cells for the same groups of non-iESR mRNAs derepressed by the *scd6Δ, edc3Δ*, or *scd6Δedc3Δ* mutations or by the *dhh1Δ, pat1Δ*, or *pat1Δdhh1Δ* mutations analyzed in (A).

bioRxiv preprint doi: https://doi.org/10.1101/2024.08.28.610059; this version posted August 28, 2024. The copyright holder for this preprint (which was not certified by peer review) is the author/funder. This article is a US Government work. It is not subject to copyright under 17 USC 105 and is also made available for use under a CC0 license.

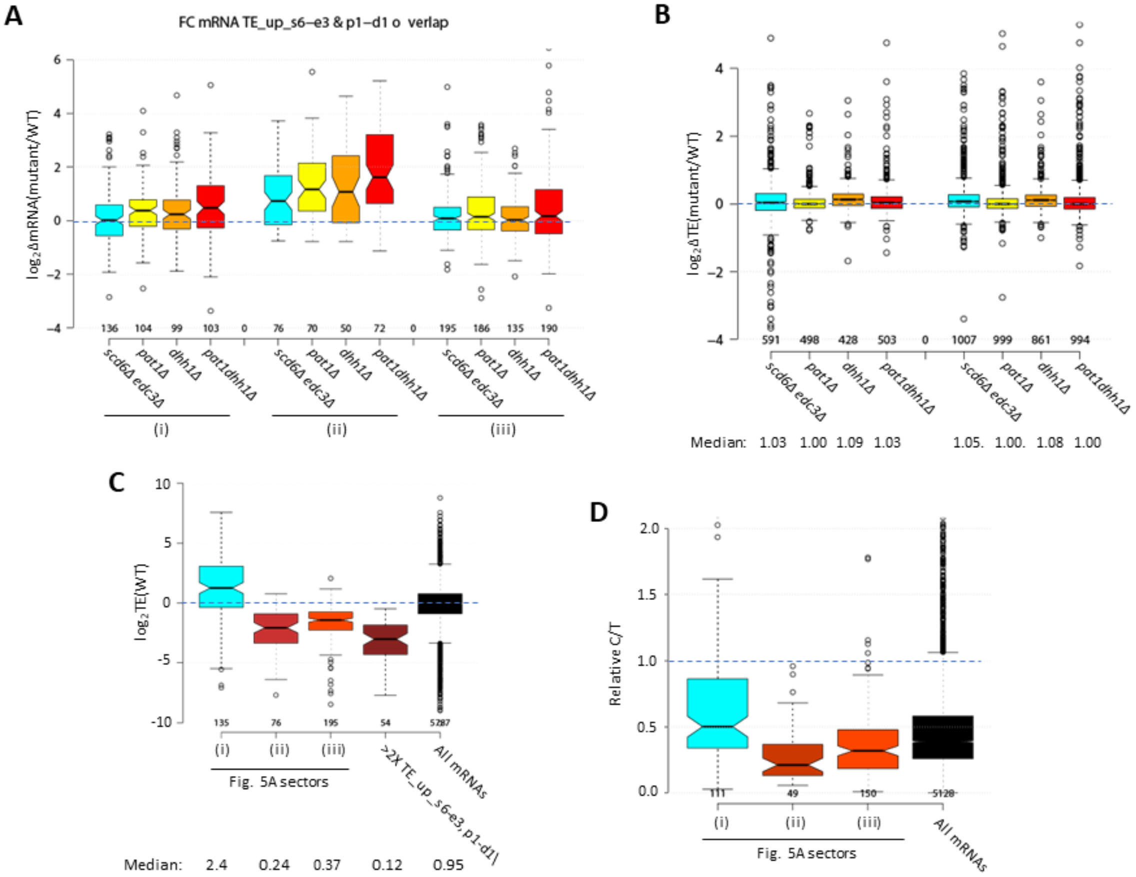

Figure S6. Properties of mRNAs translationally repressed by Scd6/Edc3 or Dhh1/Pat1. (A) Notched box-plots of $\log_2\Delta$mRNA values between the indicated mutants vs. WT for the translationally up-regulated mRNAs belonging to the specified sectors of the diagram in Fig. 5A. **(B)** Notched box-plots of $\log_2\Delta$TE values between the indicated mutants vs. WT for the mRNAs belonging to the 591 non-iESR mRNAs derepressed in abundance by *scd6Δ, edc3Δ*, or *scd6Δedc3Δ* vs. WT or the 1018 non-ESR mRNAs up-regulated by *dhh1Δ, pat1Δ*, or *pat1Δdhh1Δ* vs. WT. **(C)** Box-plot of $\log_2$ values of TE determined in WT cells for the translationally up-regulated mRNAs belonging to the specified sectors of the diagram in Fig. 5A, the 54 mRNAs showing >2-fold TE increases conferred by both *scd6Δedc3Δ* and *pat1Δdhh1Δ* mutations vs. WT, or for all mRNAs. **(D)** Box-plot of ratios of capped to total mRNA abundance in TPMs (Relative C/T) in WT cells for

bioRxiv preprint doi: https://doi.org/10.1101/2024.08.28.610059; this version posted August 28, 2024. The copyright holder for this preprint (which was not certified by peer review) is the author/funder. This article is a US Government work. It is not subject to copyright under 17 USC 105 and is also made available for use under a CC0 license.

for the translationally up-regulated mRNAs belonging to the specified sectors of the diagram in Fig. 5A, or for all mRNAs.

bioRxiv preprint doi: https://doi.org/10.1101/2024.08.28.610059; this version posted August 28, 2024. The copyright holder for this preprint (which was not certified by peer review) is the author/funder. This article is a US Government work. It is not subject to copyright under 17 USC 105 and is also made available for use under a CC0 license.

A

*591 Non-iESR mRNAs w/ abundance repressed by Edc3/Scd6**			
**Category**	**p-value**	**k**	**f**
GO Biological process			
ATP synthesis coupled proton transport [GO:0015986]	3.23E-07	10	17
mitochondrial electron transport, cytochrome c to oxygen [GO:0006123]	1.37E-06	8	12
cristae formation [GO:0042407]	5.56E-06	5	5
ATP biosynthetic process [GO:0006754]	6.56E-06	12	31
cellular cell wall organization [GO:0007047]	7.22E-06	22	89
GO Cellular Component			
mitochondrial inner membrane [GO:0005743]	5.86E-09	45	204
mitochondrion [GO:0005739]	2.60E-08	145	1072
mitochondrial respiratory chain complex IV [GO:0005751]	5.81E-08	9	12
fungal-type cell wall [GO:0009277]	3.19E-06	22	85
MIPS Functional Classification			
electron transport and membrane-associated energy conservation [02.11]	<1e-14	34	58
electron transport [20.01.15]	2.02E-10	28	83
aerobic respiration [02.13.03]	4.75E-09	25	77
energy generation (e.g. ATP synthase) [02.45.15]	3.16E-08	12	21
respiration [02.13]	8.95E-06	17	59
(ii) ***212 mRNAs translationaly repressed by Edc3/Scd6#***			
GO Biological process			
asparagine catabolic process [GO:0006530]	5.04E-06	4	5
cellular aldehyde metabolic process [GO:0006081]	1.28E-05	5	11
GO Cellular Component			
cell wall [GO:0005618]	8.85E-06	11	68
extracellular region [GO:0005576]	9.09E-06	13	95
ribosome [GO:0005840]	1.70E-05	25	310
MIPS Functional Classification			
degradation of asparagine [01.01.06.02.02]	5.04E-06	4	5
fermentation [02.16]	6.92E-06	7	24

B

*1018 Non-iESR mRNAs w/ abundance repressed by Pat1/Dhh1**			
**Category**	**p-value**	**k**	**f**
GO Biological process			
tricarboxylic acid cycle [GO:0006099]	2.87E-08	18	30
mitochondrial respiratory chain complex IV assembly [GO:0033617]	4.03E-06	10	14
GO Cellular Component			
fungal-type cell wall [GO:0009277]	3.12E-08	34	85
cell wall [GO:0005618]	9.82E-07	27	68
extracellular region [GO:0005576]	2.29E-06	33	95
mitochondrion [GO:0005739]	4.81E-06	215	1072
mitochondrial inner membrane [GO:0005743]	1.20E-05	55	204
plasma membrane [GO:0005886]	1.93E-05	83	350
MIPS Functional Classification			
tricarboxylic-acid pathway (citrate cycle, Krebs cycle, TCA cycle) [02.10]	5.86E-08	18	31
electron transport and membrane-associated energy conservation [02.11]	3.90E-07	25	58
aerobic respiration [02.13.03]	1.43E-06	29	77
(ii) ***274 mRNAs translationaly repressed by Pat1/Dhh1#***			
GO Molecular Function			
sequence-specific DNA binding [GO:0043565]	3.85E-06	21	165
GO Biological process			
cellular aldehyde metabolic process [GO:0006081]	1.88E-06	6	11
allantoin catabolic process [GO:0000256]	2.33E-06	5	7
transmembrane transport [GO:0055085]	6.78E-06	30	303
flocculation [GO:0000128]	1.40E-05	4	5
GO Cellular Component			
cell wall [GO:0005618]	3.29E-06	13	68
extracellular region [GO:0005576]	7.03E-06	15	95
plasma membrane [GO:0005886]	4.41E-05	31	350
MIPS Functional Classification			
C-compound and carbohydrate metabolism [01.05]	4.18E-07	27	223
fermentation [02.16]	3.27E-06	8	24
catabolism of nitrogenous compounds [01.02.02.09]	1.40E-05	4	5
metabolism of nonprotein amino acids [01.20.17.01]	2.52E-05	5	10
osmosensing and response [34.11.03.13]	7.03E-05	8	35
proton driven symporter [20.03.02.02.01]	7.07E-05	3	3

C

853 mRNAs with ribosome occupancy repressed by Edc3/Scd6#			
GO Biological process			
response to stress [GO:0006950]	2.57E-11	51	152
thiamine biosynthetic process [GO:0009228]	9.28E-07	11	17
mitochondrial respiratory chain complex IV assembly [GO:0033617]	1.05E-05	9	14
glycogen biosynthetic process [GO:0005978]	2.30E-05	8	12
mitochondrial electron transport, cytochrome c to oxygen [GO:0006123]	2.30E-05	8	12
GO Cellular Component			
mitochondrion [GO:0005739]	2.81E-13	227	1072
mitochondrial inner membrane [GO:0005743]	1.39E-08	56	204
extracellular region [GO:0005576]	1.45E-05	28	95
mitochondrial intermembrane space [GO:0005758]	1.60E-05	16	40
plasma membrane enriched fraction [GO:0001950]	1.78E-05	26	86
mitochondrial respiratory chain complex IV [GO:0005751]	2.30E-05	8	12
cell wall [GO:0005618]	2.43E-05	22	68
fungal-type cell wall [GO:0009277]	4.31E-05	25	85
MIPS Functional Classification			
electron transport and membrane-associated energy conservation [02.11]	1.24E-13	31	58
metabolism of energy reserves (e.g. glycogen, trehalose) [02.19]	1.81E-11	28	56
sugar, glucoside, polyol and carboxylate catabolism [01.05.02.07]	5.92E-11	34	81
stress response [32.01]	1.15E-09	50	162
aerobic respiration [02.13.03]	2.98E-08	29	77
electron transport [20.01.15]	2.01E-07	29	83
C-compound and carbohydrate metabolism [01.05]	3.82E-07	56	223
oxidative stress response [32.01.01]	4.14E-07	22	55
sugar, glucoside, polyol and carboxylate anabolism [01.05.02.04]	1.94E-06	16	35
oxidation of fatty acids [02.25]	1.67E-05	7	9
glutathione conjugation reaction [32.07.07.03]	3.56E-05	5	5
detoxification by modification [32.07.03]	0.0001015	6	8

D

1350 mRNAs with ribosome occupancy repressed by Pat1/Dhh1#			
GO Biological process			
tricarboxylic acid cycle [GO:0006099]	6.14E-09	21	30
response to stress [GO:0006950]	4.06E-06	55	152
glycogen biosynthetic process [GO:0005978]	5.45E-06	10	12
cellular response to oxidative stress [GO:0034599]	1.77E-05	29	67
GO Cellular Component			
mitochondrion [GO:0005739]	3.75E-12	305	1072
fungal-type cell wall [GO:0009277]	5.62E-12	46	85
extracellular region [GO:0005576]	1.06E-08	44	95
cell wall [GO:0005618]	1.15E-08	35	68
plasma membrane [GO:0005886]	7.34E-05	101	350
MIPS Functional Classification			
metabolism of energy reserves (e.g. glycogen, trehalose) [02.19]	8.14E-09	31	56
C-compound and carbohydrate metabolism [01.05]	1.75E-08	81	223
oxidative stress response [32.01.01]	1.05E-07	29	55
tricarboxylic-acid pathway (citrate cycle, Krebs cycle, TCA cycle) [02.10]	7.92E-07	19	31
stress response [32.01]	1.42E-06	59	162
electron transport and membrane-associated energy conservation [02.11]	1.82E-06	28	58
aerobic respiration [02.13.03]	6.14E-06	33	77
metabolism of vitamins, cofactors, and prosthetic groups [01.07]	7.04E-06	22	43
sugar, glucoside, polyol and carboxylate catabolism [01.05.02.07]	7.75E-06	34	81
osmotic and salt stress response [32.01.03]	3.27E-05	26	

Figure S7. mRNAs repressed in abundance or translation by Scd6/Edc3 or Dhh1/Pat1 are enriched for common functional categories, including Ox. Phos. proteins and cell wall

bioRxiv preprint doi: https://doi.org/10.1101/2024.08.28.610059; this version posted August 28, 2024. The copyright holder for this preprint (which was not certified by peer review) is the author/funder. This article is a US Government work. It is not subject to copyright under 17 USC 105 and is also made available for use under a CC0 license.

components. **(A-B)** Functional categories showing enrichment for genes encoding the 591 non-iESR mRNAs derepressed in abundance by *scd6Δ, edc3Δ*, or *scd6Δedc3Δ* vs. WT or the 1018 non-ESR mRNAs up-regulated by *dhh1Δ, pat1Δ*, or *pat1Δdhh1Δ* vs. WT, using color-coding to indicate related functions or cellular components. **(C-D)** Functional categories showing enrichment for genes encoding the 853 mRNAs with RPFs up-regulated by *scd6Δ, edc3Δ*, or *scd6Δedc3Δ* vs. WT or the 1350 mRNAs with RPFs up-regulated by *dhh1Δ, pat1Δ*, or *pat1Δdhh1Δ* vs. WT, conducted using the Web-based tool FunSpec and applying the Bonferroni correction and P<0.05 cutoff.

bioRxiv preprint doi: https://doi.org/10.1101/2024.08.28.610059; this version posted August 28, 2024. The copyright holder for this preprint (which was not certified by peer review) is the author/funder. This article is a US Government work. It is not subject to copyright under 17 USC 105 and is also made available for use under a CC0 license.

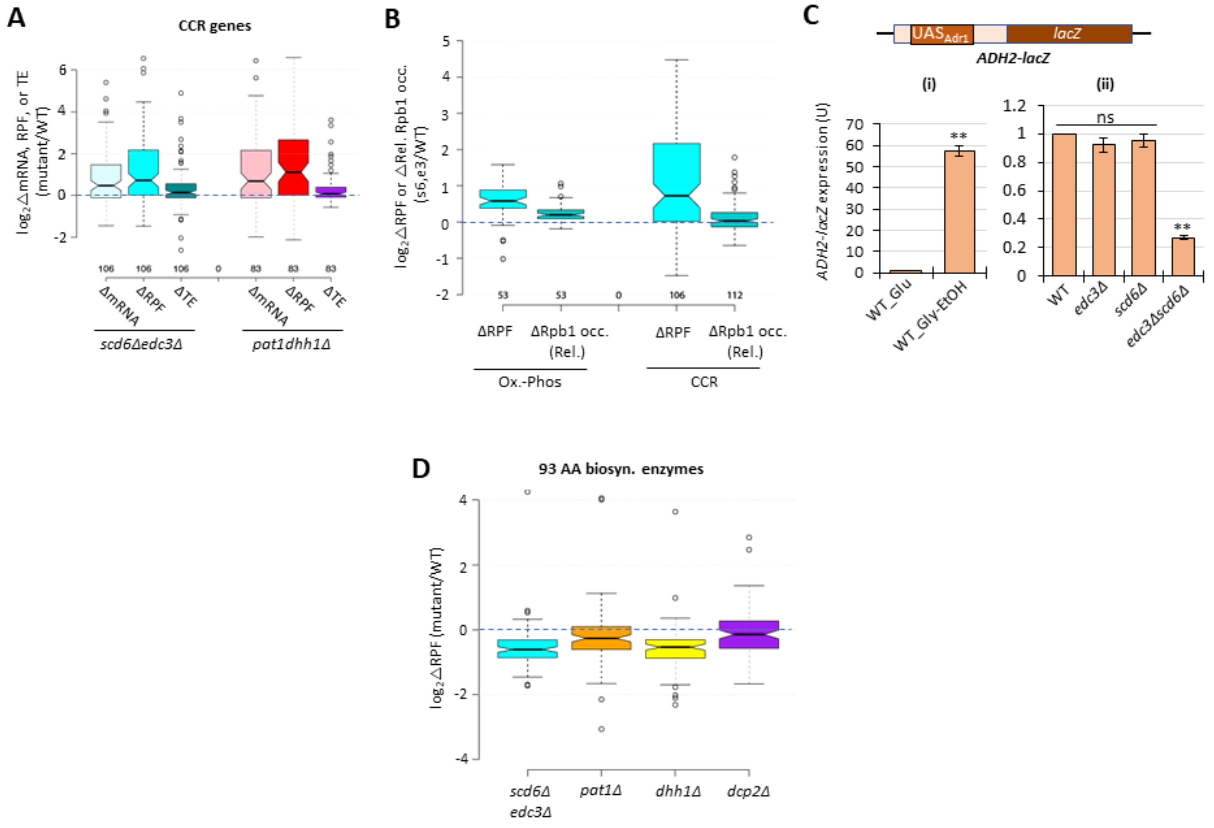

Figure S8. Scd6/Edc3 post-transcriptionally repress carbon-catabolite-repressed (CCR) genes in rich medium. (A) Log_2 changes in mRNA, RPFs, or TE conferred by the indicated double mutations vs. WT for 106 genes subject to carbon catabolite repression or activated by transcription factors Adr1 or Cat8. **(B)** Notched box-plots showing log_2ΔRPFs or log_2ΔRpb1 relative occupancies averaged over the CDSs in *scd6Δedc3Δ* vs. WT cells for the same Ox-Phos. (left) or CCR genes (right) analyzed in Figures 6A and S7A, respectively. **(C)** Expression of the *ADH2-lacZ* reporter on plasmid pLGADH2, containing the entire *ADH2* 5′ non-coding region, in the WT strain grown on SC-Ura medium containing either 2% glucose or 3% glycerol/2% ethanol as carbon sources (i), or in WT and the indicated mutant strains on SC-Ura cultured with 2% glucose (ii). For (ii), mean values of β-galactosidase activity measured for 3 biological replicates of each strain were

bioRxiv preprint doi: https://doi.org/10.1101/2024.08.28.610059; this version posted August 28, 2024. The copyright holder for this preprint (which was not certified by peer review) is the author/funder. This article is a US Government work. It is not subject to copyright under 17 USC 105 and is also made available for use under a CC0 license.

normalized to the mean value measured for WT cells. **, P-value <0.01 from student's t-test; ns, not significant. **(D)** Log_2 changes in RPFs for 93 genes encoding amino acid biosynthetic enzymes in the indicated mutants vs. WT.

bioRxiv preprint doi: https://doi.org/10.1101/2024.08.28.610059; this version posted August 28, 2024. The copyright holder for this preprint (which was not certified by peer review) is the author/funder. This article is a US Government work. It is not subject to copyright under 17 USC 105 and is also made available for use under a CC0 license.

Gene id	Gene	log_2ΔmRNA (*scd6Δ*/WT)	FDR	log_2ΔmRNA (*edc3Δ*/WT)	FDR	log_2ΔmRNA (*scd6Δedc3Δ*/WT)	FDR	Targeted by
YGL222C	*EDC1*	0.415787885	4.48E-05	0.316768752	0.257140053	1.98410287	1.86E-155	Dhh1
YGL056C	*SDS23*	0.05093765	0.764889364	-0.201352497	0.530102611	0.472035691	8.08E-10	Dhh1
YDR343C	*HXT6*	0.328862276	0.422677908	0.683203554	0.035918099	2.593164006	4.20E-28	Dhh1/Pat1
YFL014W	*HSP12*	0.731412062	0.033263692	0.885066918	0.115640255	6.847403632	0	Dhh1/Pat1

Figure S9. Synthetic genetic derepression of four Dhh1 target mRNAs on combining *scd6Δ* and *edc3Δ* mutations. RNA-seq results obtained here for the four Dhh1 target mRNAs, two of which were designated as Pat1 targets as well, identified by He et al. (2022), in the *scd6Δ, edc3Δ,* and *scd6Δedc3Δ* mutants versus WT. The log_2ΔmRNA values for each of the three mutants vs. WT are listed together with the FDR values and color-coded according to derepression ratios.

bioRxiv preprint doi: https://doi.org/10.1101/2024.08.28.610059; this version posted August 28, 2024. The copyright holder for this preprint (which was not certified by peer review) is the author/funder. This article is a US Government work. It is not subject to copyright under 17 USC 105 and is also made available for use under a CC0 license.

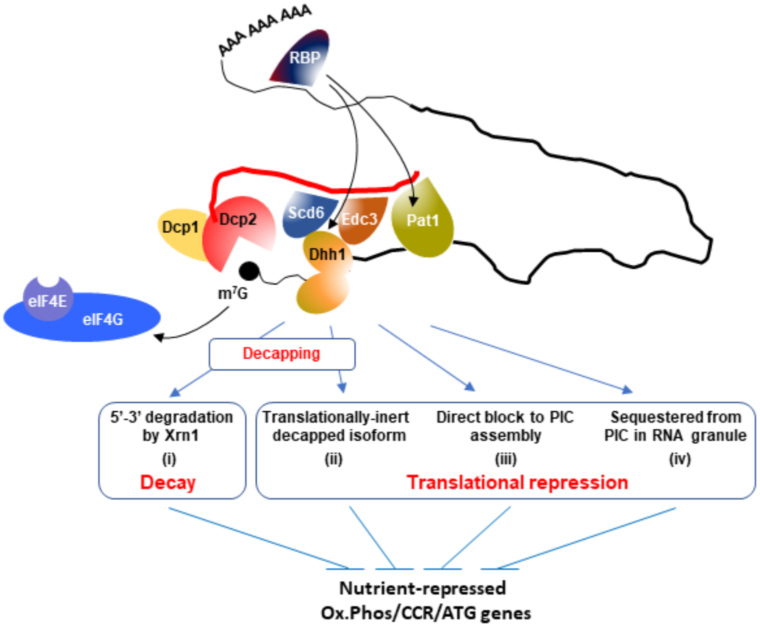

Figure S10. Hypothetical model to explain concerted repression of mRNA abundance or translation of particular mRNAs by decapping activators Scd6, Edc3, Dhh1, and Pat1. A complex of Dcp1:Dcp2 containing Scd6/Edc3, Pat1/Dhh1, or different combinations of these factors, is recruited to target mRNAs, possibly by a sequence-specific RNA-binding protein (RBP) binding to the 3'UTR. In agreement with a recent proposal (10), the recruitment of Dhh1 is enhanced by its redundant interactions with Scd6 or Edc3, which interact with the same segment of the Dcp2 CTT, whereas Pat1 is recruited independently to a distinct region of the CTT. One outcome of association of the decapping complex with an mRNA is activation of decapping with attendant 5'-3' degradation by Xrn1 occurring without detectable repression of translation (i). A different outcome is translational repression by decapping wherein degradation by Xrn1 is inefficient and decapped intermediates accumulate that cannot bind the cap-binding initiation factors eIF4E and eIF4G (depicted as subunits of eIF4F excluded from the

bioRxiv preprint doi: https://doi.org/10.1101/2024.08.28.610059; this version posted August 28, 2024. The copyright holder for this preprint (which was not certified by peer review) is the author/funder. This article is a US Government work. It is not subject to copyright under 17 USC 105 and is also made available for use under a CC0 license.

m^7G mRNA cap) and thus persist as translationally inert isoforms (ii). Alternatively, decapping may not occur and the decapping complex competes with the cap-binding initiation factors to selectively inhibit translation initiation (iii), a fate that could be favored by sequestration of the transcript in RNA granules in a manner facilitated by the decapping activator proteins (iv).

bioRxiv preprint doi: https://doi.org/10.1101/2024.08.28.610059; this version posted August 28, 2024. The copyright holder for this preprint (which was not certified by peer review) is the author/funder. This article is a US Government work. It is not subject to copyright under 17 USC 105 and is also made available for use under a CC0 license.

SUPPLEMENTARY FILES

File S1: Parallel RNA-Seq and Ribo-Seq analysis of decapping mutants, gene groups dysregulated in decapping mutants or belonging to specific functional categories, and capped/total mRNA ratios, codon optimality scores, codon protection indices, and Dhh1 occupancy values for all mRNAs. Sheets 1-7 labeled "mutant vs WT all comp" contain the processed data from Ribo-seq and RNA-seq analysis of 2 or 3 biological replicates of each strain listing the fold-changes in mRNA, RPFs, or TE between the relevant mutant vs. WT with the P-values and adjusted P-values (FDRs) assigned by DESeq2 analysis. The data for *dhh1Δ, dcp2Δ* (3)*, pat1Δ,* and *pat1Δdhh1Δ* mutants (8) were reported previously. Sheet “Gene groups_mRNA analysis” contains the lists of mRNAs (identified by systematic gene name) that are up-regulated or down-regulated in abundance by ≥1.5-fold at FDR < 0.05 in the *scd6Δ, edc3Δ,* or *scd6Δedc3Δ* mutants vs. WT, either including or excluding iESR mRNAs for up-regulated transcripts or rESR mRNAs for down-regulated transcripts, as indicated. It also lists the transcripts belonging to the three sectors of Figs. 1B-C, the iESR and rESR transcripts defined previously (11), and all of the non-iESR mRNAs up-regulated by ≥1.5-fold at FDR < 0.05 in *dhh1Δ, pat1Δ,* or *pat1Δdhh1Δ* mutants, as well as those equally up-regulated by the *dcp2Δ* mutation. Sheet “Gene groups_TE analysis” contains the lists of mRNAs that are up-regulated or down-regulated in TE by ≥1.41-fold at FDR < 0.10 in the different mutants vs. WT. Sheet “Gene groups_RPF analysis” contains the lists of mRNAs that are up-regulated or down-regulated in RPFs by ≥1.5-fold at FDR < 0.05 in the different mutants vs. WT. Sheet “Pathway gene groups” lists the genes involved in specific pathways (eg. Ox. Phos.) analyzed for changes in expression

bioRxiv preprint doi: https://doi.org/10.1101/2024.08.28.610059; this version posted August 28, 2024. The copyright holder for this preprint (which was not certified by peer review) is the author/funder. This article is a US Government work. It is not subject to copyright under 17 USC 105 and is also made available for use under a CC0 license.

or translation. Sheet “Codon Opt Scores nonESR mRNAs” lists the tRNA adaptation index (tAI), species-specific tRNA adaptation index (stAI), and the average codon stabilization coefficient (AvgCSC) for all non-ESR yeast genes (12,13). Sheet “Codon Protection Indices” lists the CPI values for all genes determined previously (14). Sheet “Capped to Total RNA ratios” lists the ratios of TPMs determined by CAGE sequencing of capped mRNAs (C) to TPMs determined by parallel RNA-seq of the same total RNA samples for 5129 genes, determined previously for *dhh1Δ* and WT cells (8). Sheet “Dhh1 occ enrich scores” lists the relative Dhh1 occupancies (enrichment scores) determined globally for yeast mRNAs by RIP-seq analysis (15) for the 3686 transcripts for which both Dhh1 enrichment score and Ribo-seq and RNA-seq data from the *dhh1Δ* vs. WT comparison (3) are available. Sheet “IGV tracks” lists the results of ribosome profiling of the *scd6Δ, edc3Δ,* and *scd6Δedc3Δ* mutants vs. WT for the genes selected for gene-browser depictions.

File S2: Relative and spike-in normalized Rpb1 occupancies from ChIP-seq analysis. Sheets 1-2 labeled "Relative occs._replicates” and “Relative occs._ Reps._averaged” contain the processed data from ChIP-Seq analysis of Rpb1 in three biological replicates of WT and *scd6Δedc3Δ* strains and the averaged data from combining the replicates, respectively, listing the relative occupancies averaged over the coding sequences for each expressed gene normalized to the average occupancy on each chromosome. Sheet “*S. pombe* norm. factor calcs,” lists the calculations of normalization factors obtained from total numbers of reads aligned to the *S. pombe* genome for each chromatin sample spiked-in with equal aliquots of *S. pombe* chromatin prior to immunoprecipitation with Rpb1 antibodies. Factors are calculated for each individual

bioRxiv preprint doi: https://doi.org/10.1101/2024.08.28.610059; this version posted August 28, 2024. The copyright holder for this preprint (which was not certified by peer review) is the author/funder. This article is a US Government work. It is not subject to copyright under 17 USC 105 and is also made available for use under a CC0 license.

replicate (col. E) or for the combined replicates for each strain (col. G). Sheets "Normalized occs._replicates" and "Normalized occs._reps._avged" lists the spike-in normalized Rpb1 occupancies calculated for each replicate or the combined replicates for each strain, respectively, calculated using the respective normalization factors for individual or combined replicates determined in the previous two sheets.

File S3: ERCC spike-in normalized RNA-Seq data. Sheet 1 lists the numbers of reads for each ERCC molecule identified in each RNA sample and calculations of the normalization factors for each sample. Sheet 2 lists the un-normalized reads for each yeast gene in each RNA sample, the ERCC-normalized reads for each sample, and the density of normalized reads for each gene calculated by normalizing for CDS lengths. Sheets 2-4 lists processed data from DESeq2, including $\log_2\Delta$ mRNA, P-value and adj. P-value determined for each gene in the indicated mutant vs. WT strain, obtained by setting the size factor to unity.

File S4: TMT-MS data. Processed data from TMT-MS analysis of *scd6Δedc3Δ* vs. WT strains. Sheet **"All proteins data" lists the normalized MS intensities of peptides for each expressed gene in three replicates each of WT (L-N) and** *scd6Δedc3Δ* (M-Q) strains and the calculated $\log_2$ fold-changes in protein expression and statistics in the *scd6Δedc3Δ* vs. WT comparison for all expressed proteins. Sheet "log2FC_protein (s6,e3 v WT)" extracts the data from columns B, Q, W, and X from the previous sheet.

bioRxiv preprint doi: https://doi.org/10.1101/2024.08.28.610059; this version posted August 28, 2024. The copyright holder for this preprint (which was not certified by peer review) is the author/funder. This article is a US Government work. It is not subject to copyright under 17 USC 105 and is also made available for use under a CC0 license.

File S5: Source data for Western blot analysis. Sheet "Fig. 6B-C Western analysis" lists averages of band intensities normalized to the loading control (Gcd6) on the same blots and the corresponding S.E.M. values calculated from the replicates for each protein analyzed in Fig. 6B-C. Sheet "Fig. 6D Western analysis" lists the Cox2 band intensities normalized to total stained proteins and the ratios of normalized Cox2 for each mutant vs. WT.

File S6: Source data for *lacZ* reporter assays. Lists the specific activities of β-galactosidase determined from three biological replicates of each strain harboring the *CYC1-lacZ* or *ADH2-lacZ* reporters analyzed in Figs. 6F and S7C, respectively.

File S7: Source data for metabolomics of polar compounds of intermediary metabolism. Sheets 1-4 list the statistical analyses of changes in metabolite concentrations in four biological replicates of the indicated four mutants vs. the WT determined in parallel for all 20 samples of metabolite extracts. Sheet 5 summarizes the $\log_2$ fold-changes in metabolite levels and corresponding P-values for all 147 metabolites detected in for each of the four mutants. Sheet 6 lists the 46 metabolites up-regulated in any two of the four mutants (analyzed in Fig. 7C), TCA cycle intermediates, and amino acids. Sheet 7 lists 93 amino acid biosynthetic genes interrogated in Fig. S8D.

File S8. Source data for glucose flux analysis. In Sheet 2 "raw intensity", the numbers indicate the signal intensities (areas under the peaks from mass spectrometry) of the metabolite listed in column A in the three biological replicates of the indicated mutant or WT strains. In the

bioRxiv preprint doi: https://doi.org/10.1101/2024.08.28.610059; this version posted August 28, 2024. The copyright holder for this preprint (which was not certified by peer review) is the author/funder. This article is a US Government work. It is not subject to copyright under 17 USC 105 and is also made available for use under a CC0 license.

subsequent sheets, raw data from Sheet 2 is collated for the different labeled isoforms of the indicated metabolite in the upper eight rows, and the proportion of the metabolite comprised of each isoform is given in the lower rows for the different samples. The "_N" labelling indicates the label addition in the metabolite, ie. _1 indicates mass addition of 1 labelled carbon, _2 indicates mass addition of 2 labelled carbons). Please let me know if you have any doubts regarding this.

File S9. Source data for measurements of cellular ATP content. Sheets 2-3 provide source data for measurements of ATP content in three biological replicates of the indicated five mutants vs. WT and the fraction of total ATP content that is eliminated by inhibiting respiration by sodium azide treatment. Sheet 4 shows the standard curve produced using pure ATP employed to determine the ATP content in cell extracts.

SUPPLEMENTARY REFERENCES

1. He, F., Li, X., Spatrick, P., Casillo, R., Dong, S. and Jacobson, A. (2003) Genome-wide analysis of mRNAs regulated by the nonsense-mediated and 5' to 3' mRNA decay pathways in yeast. *Mol Cell*, **12**, 1439-1452.
2. He, F. and Jacobson, A. (2015) Control of mRNA decapping by positive and negative regulatory elements in the Dcp2 C-terminal domain. *RNA*, **21**, 1633-1647.
3. Zeidan, Q., He, F., Zhang, F., Zhang, H., Jacobson, A. and Hinnebusch, A.G. (2018) Conserved mRNA-granule component Scd6 targets Dhh1 to repress translation initiation and activates Dcp2-mediated mRNA decay in vivo. *PLoS Genet*, **14**, e1007806.

bioRxiv preprint doi: https://doi.org/10.1101/2024.08.28.610059; this version posted August 28, 2024. The copyright holder for this preprint (which was not certified by peer review) is the author/funder. This article is a US Government work. It is not subject to copyright under 17 USC 105 and is also made available for use under a CC0 license.

4. Charenton, C., Gaudon-Plesse, C., Fourati, Z., Taverniti, V., Back, R., Kolesnikova, O., Seraphin, B. and Graille, M. (2017) A unique surface on Pat1 C-terminal domain directly interacts with Dcp2 decapping enzyme and Xrn1 5'-3' mRNA exonuclease in yeast. *Proc Natl Acad Sci U S A*, **114**, E9493-E9501.

5. Gietz, R.D. and Sugino, A. (1988) New yeast-Escherichia coli shuttle vectors constructed with in vitro mutagenized yeast genes lacking six-base pair restriction sites. *Gene*, **74**, 527-534.

6. Sloan, J.S., Dombek, K.M. and Young, E.T. (1999) Post-translational regulation of Adr1 activity is mediated by its DNA binding domain. *J Biol Chem*, **274**, 37575-37582.

7. Forsburg, S.L. and Guarente, L. (1989) Identification and characterization of HAP4: a third component of the CCAAT-bound HAP2/HAP3 heteromer. *Genes Dev*, **3**, 1166-1178.

8. Vijjamarri, A.K., Gupta, N., Onu, C., Niu, X., Zhang, F., Kumar, R., Lin, Z., Greenberg, M.L. and Hinnebusch, A.G. (2023) mRNA decapping activators Pat1 and Dhh1 regulate transcript abundance and translation to tune cellular responses to nutrient availability. *Nucleic Acids Res*.

9. O'Duibhir, E., Lijnzaad, P., Benschop, J.J., Lenstra, T.L., van Leenen, D., Groot Koerkamp, M.J., Margaritis, T., Brok, M.O., Kemmeren, P. and Holstege, F.C. (2014) Cell cycle population effects in perturbation studies. *Mol Syst Biol*, **10**, 732.

10. He, F., Wu, C. and Jacobson, A. (2022) Dcp2 C-terminal cis-binding elements control selective targeting of the decapping enzyme by forming distinct decapping complexes. *Elife*, **11**.

bioRxiv preprint doi: https://doi.org/10.1101/2024.08.28.610059; this version posted August 28, 2024. The copyright holder for this preprint (which was not certified by peer review) is the author/funder. This article is a US Government work. It is not subject to copyright under 17 USC 105 and is also made available for use under a CC0 license.

11. Gasch, A.P., Spellman, P.T., Kao, C.M., Carmel-Harel, O., Eisen, M.B., Storz, G., Botstein, D. and Brown, P.O. (2000) Genomic expression programs in the response of yeast cells to environmental changes. *Mol Biol Cell*, **11**, 4241-4257.

12. Presnyak, V., Alhusaini, N., Chen, Y.H., Martin, S., Morris, N., Kline, N., Olson, S., Weinberg, D., Baker, K.E., Graveley, B.R. *et al.* (2015) Codon optimality is a major determinant of mRNA stability. *Cell*, **160**, 1111-1124.

13. Radhakrishnan, A., Chen, Y.H., Martin, S., Alhusaini, N., Green, R. and Coller, J. (2016) The DEAD-Box Protein Dhh1p Couples mRNA Decay and Translation by Monitoring Codon Optimality. *Cell*, **167**, 122-132 e129.

14. Pelechano, V., Wei, W. and Steinmetz, L.M. (2015) Widespread Co-translational RNA Decay Reveals Ribosome Dynamics. *Cell*, **161**, 1400-1412.

15. Miller, J.E., Zhang, L., Jiang, H., Li, Y., Pugh, B.F. and Reese, J.C. (2018) Genome-Wide Mapping of Decay Factor-mRNA Interactions in Yeast Identifies Nutrient-Responsive Transcripts as Targets of the Deadenylase Ccr4. *G3 (Bethesda)*, **8**, 315-330.
